# Development of a stereo dip-coating system for fabrication of tube-shaped blood vessel models

**DOI:** 10.1038/s41598-020-63718-w

**Published:** 2020-04-24

**Authors:** Yasutomo Shimizu, Simon Tupin, Chihaya Kiyomitsu, Ko Kitamura, Kazuto Takashima, Makoto Ohta

**Affiliations:** 1 Institute of Fluid Science, Tohoku University, 2-1-1, Katahira, Aoba-ku, Sendai, Miyagi Japan; 2 Graduate School of Biomedical Engineering, Tohoku University, 6-6, Aramaki-aza-aoba, Aoba-ku, Sendai, Miyagi Japan; 3 Graduate School of Life Science and Systems Engineering, Kyushu Institute of Technology, 2-4, Hibikino, Wakamatsu-ku, Kitakyushu, Fukuoka Japan

**Keywords:** Biomedical engineering, Mechanical engineering

## Abstract

Tube-shaped blood vessel models that mimic their geometries and mechanical properties can deliver reliable and realistic behavioral information such as deformation and rupture during procedures such as insertion of medical devices. Thickness of vessel walls is an important parameter for fabricating the blood vessel models owing to their strong influence on the model behavior, especially during deformation. The dip-coating method is used to fabricate blood vessel models; however, non-uniform wall thicknesses are observed using this method. This study aimed at finding the characteristics of stereo “angular control dip-coating” (ACDC) system to develop a dip-coating system that can produce tubular models with uniformed wall thickness. The system developed here enables an observation of the substrate behavior from two different views. The conditions of dip-coating used in this study produce 1.36–1.82 mm in the maximum and 0.188–0.435 mm in minimum wall thickness and the fabricated walls cover the realistic range of carotid arterial dimensions. The characteristics of the ACDC system indicate that ACDC is effective for fabricating the uniform wall thickness particularly in the strong curved parts.

## Introduction

Diseases related to the blood vessels are generally treated using intravascular treatments. Advanced intravascular procedures demand trainings for improving the technical skills of new medical doctors and also for evaluating the safety of new medical devices^1–3^. Training that use *in vitro* models is expected to improve the current educational awareness among healthcare practitioners owing to their high performance in representing realistic blood vessel characteristics, which also enable to decrease the number of animal test.

Poly(vinyl alcohol) hydrogel (PVA-H) materials have been used to develop such *in vitro* models because PVA-H can closely mimic both geometries and mechanical properties of human tissues, including blood vessels^4^. Additionally, the high transparency of the PVA-H allows direct optical observations without requiring the use of X-ray imaging techniques.

The geometries of PVA-H-based blood vessel models can either be categorized as box-or tube-shaped^4^. The box-shaped models are easier to fabricate and maintain compared with the tube-shaped models. However, it is difficult to observe blood vessel rupture and realistic deformations using this model due to their thickness. In contrast, the tube-shaped models represent realistic geometries and mechanical properties can produce a more realistic behavior, such as deformation and rupture, during the insertion of medical devices.

Three techniques namely, painting, cast molding, and dip-coating, are used for fabricating film and hydrogel models^5,6^. The painting method is a manual-handling technique that requires high technical skills for fabricators to accurately reproduce complex geometries including wall thickness. The cast molding method can reproduce uniform wall thickness, as based on the mold, however, there is no flexibility that allows changes in wall thickness. Recently, 3D printing technique has demonstrated potential for fabrication of realistic blood vessel geometries. However, this technique faces several problems such as processing accuracy and limitations of fabricating geometries^7,8^.

The dip-coating, which is widely adopted in industrial settings, can fabricate films with simple geometries^6,9–12^. This method is mainly used for flat geometries, such as cylinder and plate, and nowadays, it is expanded to complex geometries including curved surfaces or loop structures as well^13–15^. The wall thickness can be controlled by the viscosity of the solution and the withdrawal speed of the substrate. In addition, the angle between the substrate and the solution plays an important role for achieving uniform wall thickness. The dip-coating method is also adopted for the fabrication of tube-shaped blood vessel models, and several dip-coating systems for such fabrications have been developed^16–19^. In order to render a uniform wall thickness after dip-coating, the spinning system was additionally mounted on the dip-coating systems. Although the influence between the methods on the surface was reported^20–23^, the difference can be shown in nano-scale level, and its effect on the model usage is very little. In spite of this development, non-uniform wall thicknesses and cracks are still observed in the fabricated vessel models because the spinning control is difficult to cover partially in substrate geometries. To solve this problem, a stereo dip-coating system would be useful because it would enable to control of the substrate angle using two different views based on the substrate geometry during dip-coating. However, few fabrication methods have been established that pre-consider 3D geometries. This study aimed to find the characteristics of stereo “angular control dip-coating” (ACDC) system using substrates with straight and curved parts to develop a dip-coating system that can produce tubular models with uniformed wall thickness.

First, the influence of the angle between the substrate and the solution surface on the wall thickness was analyzed in this study. Later, the dip-coating was performed using the developed system and distribution of the wall thickness was compared between the angular control dip-coating and no ACDC models using the images acquired by a micro computed tomography system (micro CT).

## Experimental Methods and Materials

### Materials

PVA powder (JF-17, DP = 1700, SV = 99 mol%, Japan Vam & Poval Co. Ltd, Japan) was dissolved in a solvent mixture of distilled water and DMSO (20/80 w/w, Toray Fine Chemicals Co. Ltd, Japan). Concentration of PVA in solvent was adjusted at 10–15 wt% because this concentration reportedly resulted in realistic mechanical properties of blood vessels in PVA-H blood vessel models^24^. In this study, the solution was prepared at 10 wt% concentration similar to the previous study in 1-directional dip-coating and at 15 wt% for representing realistic blood vessel stiffness in stereo dip-coating^16,24^.

The PVA solution was stirred for 2 h at 100 °C and allowed to cool down to 45 °C. The PVA solution is known to behave as a non-Newtonian fluid whose viscosity can be changed depending on concentration and solution temperature^7,12^. In this study, solution viscosity was measured under 30 Hz of frequency using a static viscometer (SV-10, A&D Instruments Ltd., UK). The viscosity of the solution at 45 °C was 1.75 Pa·s at 10 wt% and 10.5 Pa·s at 15 wt% concentration. The solution at 45 °C was poured into an acrylic PVA solution bath. After dipping of substrates, the solution was stored at −30 °C for 24 h to promote gelation of the PVA solution.

### Substrate preparation

**Figure 1:**
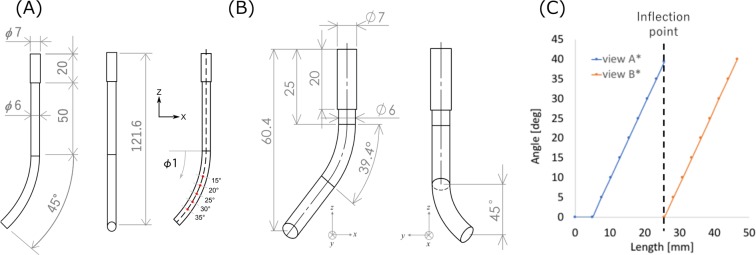
Geometries of the substrate. (**A**) ideal geometry and measuring points of wall thickness for 1-directional dip-coating, (**B**) ideal geometry for stereo dip-coating, (**C**) the relationship between angles at measuring points from two views and length from the top in the centerline. Left panel shows the frontal view (view A*) and right panel shows the lateral view (view B*). The drawings are not to scale (unit: mm).

Two ideal geometries for substrates were fabricated using ABS filament by a 3D printer (M200, Zortrax S.A., Poland) (Fig. 1). The ideal cylinder model was prepared for 1-direction dip-coating, its dimensions were: 6 mm diameter, 45.0° curvature and 1/70 mm^−1^ curvature radius. The top 20 mm had a diameter of 7 mm, and this part formed a grabbed part of the dip-coater (Fig. 1(A)). Another ideal cylinder model was prepared for stereo dip-coating, and it had a grabbed part and two curvatures with 6 mm of diameter each. The angles of curvature were 39.4° and 45.0° with 1/30 mm^−1^ of curvature radius (Fig. 1(B)). To contextualize the entire model, the relationship between the angle from the two views and length from the top in the centerline is shown in Fig. 1(C). Three substrates were prepared in each geometry. Tortuosity is a geometrical parameter for clarification of the realistic vessel geometry and this parameter can be calculated as the ratio of the curved length of the blood vessel to the straight-line distance between the two endpoints, as shown in Eq. (1).

1 $${T}_{0}=\frac{{L}_{c}}{{L}_{s}}$$

here, *T*_0_*, L*_*c*_*, L*_*s*_ are tortuosity, curved length, and distance of the straight line, respectively^25^. The tortuosity of the frontal view (view A*) is 1.026 and that of lateral view (view B*) is 1.020. These values are in the range of the common carotid artery between 1.10 ± 0.1^25^. The substrates were dyed using a black lacquer to recognize themselves in the program using image thresholding.

### Development of an angular control system

#### Concept

**Figure 2:**
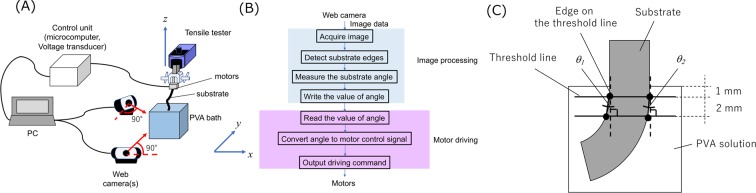
Angular control dip-coating (ACDC) system. (**A**) Outline image of the system, right panel, (**B**) Working process of the program, (**C**) Mechanism of the detection at the edges of substrate. The edge is detected from the binarization of the image.

Figure 2 depicts the outline concept image of ACDC system. This system can continuously measure the angle between the substrate and the liquid surface. Based on accumulated data, the substrate can also be continuously rotated to maintain a 90° angle. The entire process is controlled by a program code, which consists of image processing and motor control (written in LabVIEW, National Instruments, USA). The substrate behavior is controlled by a tensile tester (EZ-S, Shimadzu Co., Ltd., Japan) for pulling up through the Z-axis and motors for rotative control. Each motor can work independently based on the feedback data from the web camera. The substrate was pulled up at a withdrawal speed of 50 mm/min in each experiment based on the previous study^16^.

Substrate recognition was performed using image thresholding based on the gray-scale values obtained from the web camera. The camera was set parallel with the side planes of the acrylic PVA bath. The signal frequencies of camera acquisition and servo motor output were both set at 2 Hz. A sheet of white paper was attached behind the transparent acrylic bath for the PVA solution to define the white value on the display. Two threshold lines were drawn at a 2 mm distance and 1 mm below the surface of PVA solution, and four points at the edges of the substrate were recognized by the gray-scale value designated as a threshold (Fig. 2(C)). The control angle was defined as the average of the angle of the left side, *θ*_1_, and on the right side, *θ*_2_.

#### Dip-coating theory

**Figure 3:**
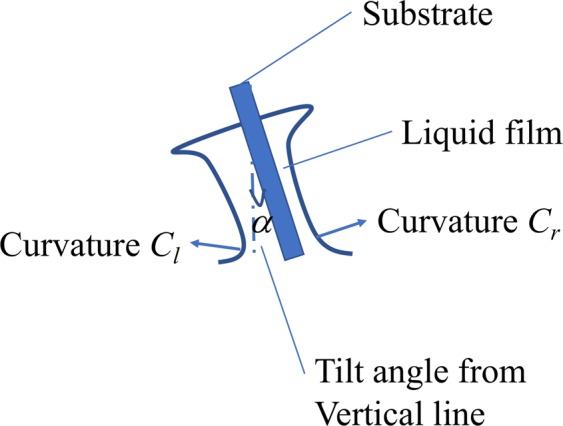
Influence of the substrate angle on the wall thickness. This figure is edited from ref. ^27^.

Angular control is an important factor for fabricating vessel models with uniform wall thickness. It can be explained from the curvature radius of meniscus of the solution, as described in Eq. (2) for the left side curvature *C*_*l*_ and Eq. (3) for the right-side curvature *C*_*r*_^26^ (Fig. 3).

2 $${C}_{l}^{-1}=\kappa \sqrt{2(1+\,\sin \,\alpha )}\,(0\le \alpha \le \frac{\pi }{2})$$

3 $${C}_{r}^{-1}=\kappa \sqrt{2(1-\,\sin \,\alpha )}\,(0\le \alpha \le \frac{\pi }{2})$$

$\kappa$ and $\alpha$ represent the inverse of capillary length and the tilting angle from the vertical line of the liquid surface in these equations. The angle $\alpha$ should be maintained at 0° during dip-coating for uniform wall thickness because this difference in curvature can produce a non-uniform wall thickness.

#### 1-directional dip-coating and angular control on the wall thickness

To perform 1-directional dip-coating, a stepping motor (CRK513PB-H50, Oriental motor Co., Ltd, Japan) and a web camera (CMS-V20SETSV, Sanwa supply Inc., Japan) were prepared for rotative control around *y* axis and image capture. The image size was 640 × 480 pixels (124.9 × 168.6 mm). The control unit consisted of a computer, a motion controller (PXI-7330, National Instruments, USA), a terminal block (UMI-7664, National Instruments, USA), and a motor driver (CRB5103PB, Oriental motor Co., Ltd, Japan). Dip-coating was performed for three substrates with the geometry depicted in Fig. 1(A) and the averaged wall thickness was measured. The measurement method is described in section 4.1.

#### Stereo dip-coating

To perform stereo dip-coating, the number of motor and web camera needed to be increased. First, two web cameras were prepared for a 2-directional observation of the substrate behavior (C930e, Logitech, Switzerland). One camera observed the substrate from the front-side of the tensile test and the other one can observe from the lateral-side. In this study, the image size was 1024 × 576 pixels (53.3 × 30.0 mm) and the camera was operated at a 4× zoom.

Two servo motors (DS215MG, ver. 3.0, KST servo, Germany) were also prepared to control the substrate using the images obtained from the web cameras. Each motor could work independently at 1200°/s of the rotation speed based on the feedback data from the web cameras around *x* and *y* axes. The angular control signals were sent to the motors via a microcomputer (Arduino Uno Rev. 3, Arduino SRL, Italy), which was controlled by LabVIEW. Dip-coating was performed for three substrates with the geometry depicted in Fig. 1(B) and the averaged wall thickness was measured. The measurement method is described in section 4.2.

### Evaluation of the method

#### 1-directional dip-coating and influence of the angular control on the wall thickness

First, 1-directional dip-coating without angular control was performed to confirm the influence of the difference in angles between the substrate and the solution surface on the wall thickness. After dip-coating and gelation of the PVA solution, micro CT images were acquired by a micro CT (ScanXmate-E080T, D160TSS105, Comscantecno Co. Ltd., Japan) to measure the wall thickness of the model. The X-ray conditions used for the image acquisition were: voltage 80–100 kV and current 100 $\mu$A. The parameters used for image resolution were as follows: magnification ratios of 2.12, spatial resolutions of 14.6–27.5 µm/pixel, and an image acquisition rate of 1800 during a 360° rotational acquisition. The detector in the micro CT imaging system had a maximum resolution of 1856 × 1472 pixels (5 mm/px). After image acquisition was performed, the images were reconstructed using an image processing software (cone CT express, White Rabbit Corporation, Japan). Three-dimensional (3D) images were developed using the reconstructed images. The thicknesses of the PVA-H models were measured every 5° from 15° to 40° of the curvature angle *ϕ*_1_ using the binarized cross-sectional images. The thickness was measured every 90° in each cross section. The binarization method will be explained in the latter section 4.2.

Using this dip-coating method, a dip-coating with angular control was also performed to compare with the results of no ACDC. The thicknesses of the PVA-H models were measured every 5° from 15° to 40° of curvature angle. A box plot was drawn to statistically assess the effectiveness of ACDC in terms of the uniformity of wall thickness in the whole models using wall thickness of inside and outside in every curvature angle. In addition, Welch’s *t*-test was also performed to evaluate the uniformity in the straight/slight curved part and the strong curved part in the substrates. The *t* and *p* values were calculated and evaluated using the averaged wall thickness of the inside and the outside for 15° and 20° of the straight/slight curved part and for 25°– 40° of the strong curved parts in three models.

#### Stereo dip-coating

**Figure 4:**
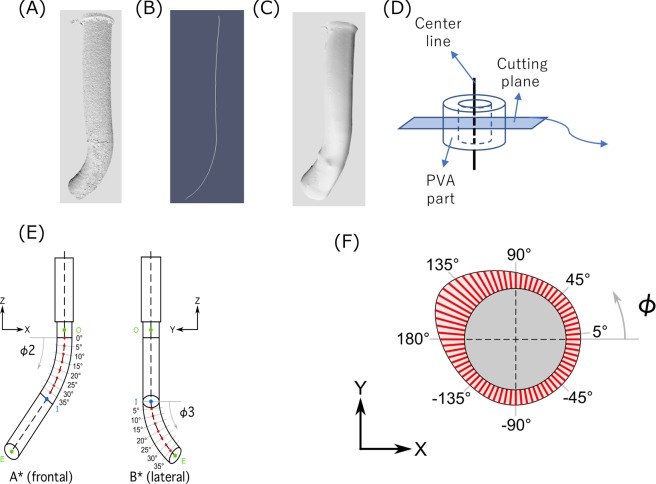
Measurement process of wall thickness in the PVA-H model. (**A**) Substrate geometry from micro CT image, (**B**) centerline from the substrate geometry, (**C**)PVA-H geometry from micro CT image, (**D**) getting the segment using the PVA-H geometry (**C**) and vertical cutting planes of the centerline (**B**), (**E**) Measuring points of the cutting planes in the substrate for stereo dip-coating in Fig. 1 (**B**) (O; original point, E; end point, I; inflection point), (**F**) wall thickness measurement on the segment of the cutting planes to evaluate the uniformity. A* and B* indicate the front and lateral view points in each curve and the numbers indicate the angle in the curves.

Stereo dip-coating was performed using the developed system and the model thickness was measured using the micro CT images to evaluate the uniformity of the wall thickness. After dip-coating and gelation of the PVA solution, micro CT images were acquired by a micro CT to measure the wall thickness of the model. The X-ray and imaging conditions were identical to those in section 4.1. PVA-H and substrate images were reconstructed from the original micro CT images. First, the substrate images were obtained to establish its stereolithography (STL) data to get its centerline because this centerline lies non-coplanar. The CT image binarized using two image processing softwares (Fiji ver. 2.0, a distribution of ImageJ, National Institute of Health, USA; Horos ver. 2.4.0, a distribution of Osirix, Horos project, USA). The window value of the images optimized using the auto bright/contrast adjustment function in Fiji and the optimized images binarized with the threshold region between 100 and 150. The STL data was obtained from these binarized images. The centerline of the substrates was acquired using another software (VMTK 1.4.0, OROBIX, Italy), and the vertical cutting planes to the centerline were obtained every 5° similar to that in the 1-directional dip-coating method to measure the wall thickness [Fig. 4(A) through (D)]. Each location of the cutting planes is shown in Fig. 4(E). In this figure, *ϕ*_2_ and *ϕ*_3_ mean the angles of view A* and view B*, respectively. Next, the PVA-H images were obtained to measure the thickness. The binarization and obtaining STL data were the same process of the substrate images but the threshold for the binarization was over 150. The wall thickness was measured every 5° in each cutting plane image, as shown in Fig. 4(F). In this figure, the angle *φ* is the rotation angle. The wall thickness distribution is also illustrated by a color contour map. The drawing of the box plot and *t*-test were also performed following the evaluation of 1 directional dip-coating. In this substrate, the straight/slight curved part was defined before the inflection point (27.5 mm) and the strong curved part was defined after the inflection point for *t*-test.

## Results

### 1-directional dip-coating and analysis of the influence of angular control on wall thickness

**Figure 5:**
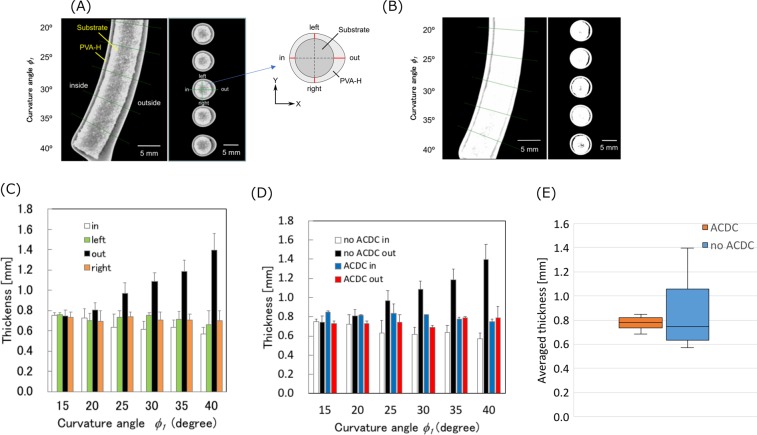
Micro CT images and thickness of the PVA-H tube-shaped models deposited on the curvature substrate. Each experiment was performed thrice (n = 3). (**A**) micro CT images of no ACDC, (**B**) micro CT images with 1-directional angular control, (**C**) averaged thickness of no ACDC (mean + SD), (**D**) averaged thickness of 1-directional ACDC and no ACDC (mean + SD), (**E**) boxplots of averaged wall thickness in whole section (mean ± SD). In (**A,B**), the left panel shows images of the vertical-sectional view and the right panel shows images of the cross-sectional view. The thickness was measured every 90° (in, out, left, and right) in each cross section.

Figures 5(A,B) show micro CT images of the PVA-H tube-shaped models with the substrates and Fig. 5(C,D) show the thickness of the PVA-H on the curvature substrate. In absence of angular control, uniformity of wall thickness was maintained at small curvature angles at 15°. In contrast, the outer thickness increased with increasing angle of curvature. In case of the curvature angles from 20° to 40°, the outer thickness is higher than the inner and the % error in measurement of the wall thickness between inside and outside is 41.7%. In addition, PVA-H distributed in a concentric pattern, except on the outside when the curvature angles were higher than 25°, which indicated that the non-uniformity of wall thickness appeared locally in this angle domain. In the presence of angular control, the difference in inner and outer wall thickness was much smaller at curvature angles from 20° to 40° compared to that in absence of angular control, and the % error in measurement of wall thickness at 40° was 1.27%. Figure 5(E) represents boxplots of the average wall thickness in the whole section. The differences between the maximum and minimum values are 0.16 mm of the ACDC model and 0.83 mm of the no ACDC model; the interquartile ranges are 0.086 mm of the ACDC model and 0.42 mm of the no ACDC model. In addition, the standard deviations of the model thickness are 0.049 mm of the ACDC model and 0.26 mm of the no ACDC model. Every parameter of the ACDC model is lower than that of the no ACDC model, and these results indicate that ACDC can produce a statistically low error variation of the parameters. This low error variation refers to a high uniformity of the wall thickness. Therefore, these results indicate that the proposed ACDC model can produce uniform wall thickness.

The absolute *t* value of the straight/slight curved parts is 0.18 (*p* > 0.05) and the *t* critical value (two-sided) is 2.26. The *t* value is lower than the critical value and this result indicates there is no significant difference between the ACDC model and the no ACDC model.

In contrast, the *t* value of the strong curved parts is 3.26 and this is higher than 2.08 of the critical value (two-sided) and this result indicates there is a significant difference between the ACDC model and the no ACDC model. This *t*-test follows that this ACDC method contributes to the effectiveness of the wall thickness uniformity in the strong curved part as confirmed in Fig. 5(C,D).

### Stereo dip-coating to evaluate the accuracy of the developed system

**Figure 6:**
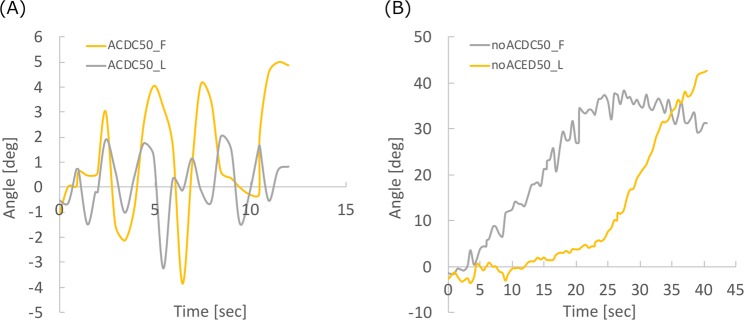
Substrate angle from the vertical line during pull-up to evaluate the ACDC behavior. The angle range on the vertical axes should be different based on the stability of the motors. ((**A**) with ACDC, (**B**) no ACDC, F; frontal view of the substrates, L; lateral view of the substrates).

**Figure 7:**
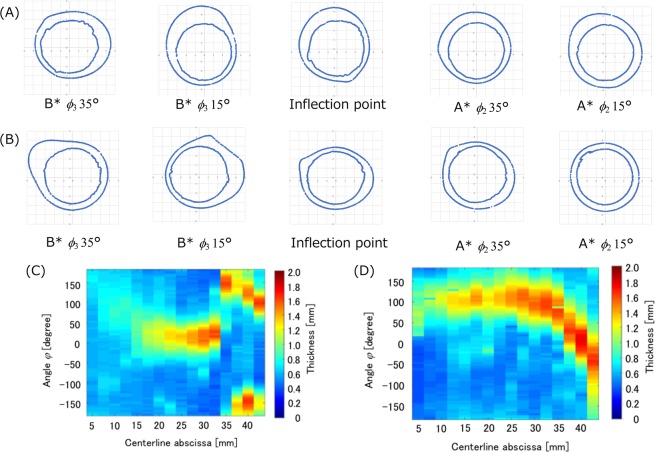
Example of cutting plane images in the substrates to measure the wall thickness on the cutting planes and to evaluate uniformity of wall thickness. (**A**) ACDC substrate, (**B**) no ACDC substrate, (**C**) Color contours of ACDC model for the relationship between wall thickness and centerline, (**D**) Color contours of no ACDC model for the relationship between wall thickness and centerline. The scales in (**A**,**B**) are 1 mm square each.

**Figure 8:**
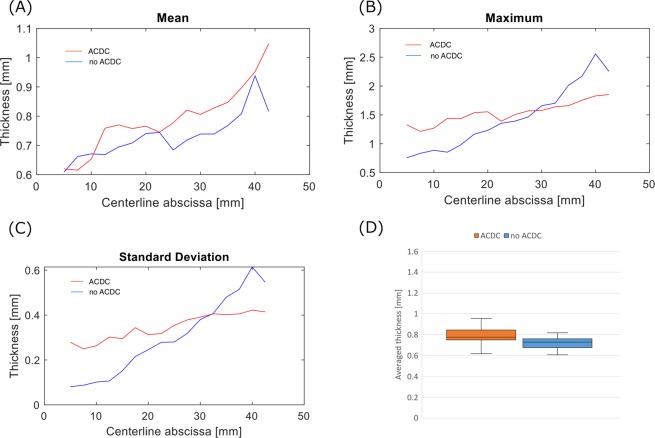
Distribution of wall thickness in each cutting plane. Each experiment was performed thrice (n = 3) and the averages of the results were plotted. (**A**) mean wall thickness, (**B**) maximum wall thickness, (**C**) standard deviation of the wall thickness. (**D**) boxplots of averaged wall thickness in whole section.

Figure 6 shows the angles of the substrate from the vertical line for comparing the effect of ACDC. Positive and negative angles indicate the rotative direction of the motors (positive; clockwise, negative; counter clockwise). The ACDC can control the angles (within 90° ± 5°) while pull-up. The angle in case of no ACDC substrate increased according to substrate geometry. This confirmed that the developed system was capable of maintaining an angle of almost 90° between the substrate and the liquid surface. Figure 7 shows an example of cutting plane images drawn from the micro CT images and color contours of the wall thickness distribution. The color contours in Fig. 7(C,D) indicate that the thickened area varies depending on the usage of ACDC and this result follows the theory described in section 3.2. Figure 8 shows the averaged distributions of wall thickness in each cutting plane in the substrate. Figure 8(A) shows the mean wall thickness of the models. The mean wall thickness of both models is 0.6–1.1, and ACDC models tend to produce walls of higher thickness than no ACDC models. Figure 8(B) shows the average of the maximum wall thickness in the cutting plane in the ideal models and the range of the wall thickness was 1.36–1.82 mm. The distributions of standard deviation (SD) are indicated in Fig. 8(C). In the ACDC models, the SD values lie between 0.267 and 0.422 mm in the entire region. In contrast, the values lie between 0.0643 and 0.607 mm in the no ACDC models. The wall thickness in no ACDC models can be represented uniformly in the straight/slight curved part, while those in the strong curved part were non-uniform. The dimension of the non-uniformity was larger than the ACDC models at the same cutting plane. Therefore, a comparison of both models indicated that the no ACDC model had a larger dimension of the non-uniformity.

Figure 8(D) represents boxplots of the average wall thickness in the whole section. The observations noted were as follows. Differences between the maximum and minimum values: the ACDC model 0.34 mm, the no ACDC model 0.207 mm; the interquartile ranges the ACDC model are 0.095 mm, the no ACDC model, 0.088 mm; the standard deviations of the model thickness: the ACDC model are 0.11 mm, the no ACDC model 0.077 mm. The absolute *t* value of the straight/slight curved parts is 4.21 (*p* < 0.05). This value is higher than 2.20 of the *t* critical values (two-sided). Moreover, the absolute value of the strong curved parts is 2.92 (*p* < 0.05) and this value is also higher than 2.45 of the *t* critical values (two-sided).

Both *t* values are higher than the critical values and these results indicate there are significant differences between the ACDC model and the no ACDC model. The non-uniform thickness is built in the straight/slight curved part is and the uniformity in the strong curved part is improved, as shown in Figs. 7 and 8. These *t*-tests follow that this ACDC method affects the wall thickness uniformity. The detail is explained in the following Discussion.

## Discussion

In this study, we developed a stereo angular control dip-coating system to improve the fabrication control of wall thickness in blood vessel models. As shown in Fig. 6, the substrate angle from the surface of the PVA solution was indicated by a stable value within 5° regardless of the pull-up speed, while ACDC was performed. In contrast, the angle increased in accordance with pull-up. This indicated that the motors in the developed system can control the substrates precisely within 5° angles based on images obtained from the cameras.

The solution and the pull-up speed *U* can theoretically influence on the wall thickness as shown in Eq. (4)^28–30^.

4 $${h}_{0}=T\sqrt{\frac{\mu U}{\rho g}}$$

In this equation, *h*_0_, *T*, $\mu$, $\rho$, and *g* represent the wall thickness, the dimensionless thickness of the deposited film, the viscosity of the solution, the density of the solution, and gravity acceleration, respectively. The dimensionless thickness includes several parameters and the relationship between the detail of this parameter and the fabrication of wall thickness can be referred from the ref. ^28^ through^28^. To consider the rotational effect in dip-coating, the angular velocity *ω* can be included as the velocity in z-direction component. The practical speed in z-direction (*U’*) can be substituted to *U* in Eq. (4).

5 $$U{\prime} =U+r\omega \,\sin \,\alpha $$

In this equation, *r*, $\omega$, and $\alpha$ represent the distance between the rotational center and the substrate on the liquid surface in the PVA bath, angular velocity of the motor, and the tilting angle of the substrate, respectively. The local pull-up speed is variable in real time because the angular velocity $\omega$ can change based on substrate geometry during dip-coating. This change in pull-up speed may produce the difference of wall thickness among cutting planes.

The wall thickness can be higher on the outside of the curvature in no ACDC models compared with the inside in ACDC models, as shown in Fig. 5(A) through Fig. 5(D). This conforms with Eqs. (2) and (3) described above. In addition, the statistical analysis shown in Fig. 5(E) indicates that the uniformity of the whole ACDC model is higher than that of the no ACDC model. This result suggests that the proposed concept and method can fabricate uniform wall thickness. High viscosity and low weight (density) solution can promote uniform wall thickness in terms of the fluid dynamics, as shown in Eq. (4). However, there is a correlation between the viscosity of the solution and the stiffness of the hydrogel^7^. Therefore, there is a limited range of selection of the viscosity for an artery model and this paper is a feasibility test of dip-coating on the fabrication of the tubular arterial models.

The results of the stereo dip-coating show that the ACDC model has the higher uniformity than no ACDC model at the 30 mm of the length and the lower part, whereas the straight/slight curved part in no ACDC model has high uniformity than ACDC model. The motor control can rotate the substrate during the dip-coating, as shown in Figs. 7 and 8. The rotation can produce the dripping and change the flow direction of the PVA solution through the substrate due to gravity and it will build the non-uniform wall thickness^31^. The flow direction change based on the dripping is not included in Eq. (4). This can be why the straight/slight curved part in no ACDC model has higher uniformity than ACDC model. The comparison of the SDs in Fig. 8(C) reveals that the no ACDC model at a low angle has lower SDs than the ACDC model, which indicates that the no ACDC model in low angle has a higher uniformity.

The stereo dip-coating models also have the non-uniformity, as shown in Figs. 7 and 8. The stereo dip-coating system has two rotation axes, and the rotations work independent of each other. This independency has an influence on the instability of the rotation axes and on the noise of the behavior, as shown in Fig. 6. Therefore, this instability also causes the non-uniformity indicated in Figs. 7 and 8. This independency and the instability would be solved by developing of a correction algorism or a hardware device. The ACDC model has the higher uniformity than no ACDC model at the 30 mm of the length and the lower part, as shown in Figs. 7 and 8. This result indicates the curvature in the substrate is more effective to fabricate the model than gravity. The fabricated models by the developed ACDC system can be reproduced in this region using the adopted dipping condition, as shown in Fig. 8(B,C). However, no ACDC model has extremely thicker part in the bottom.

The *t*-test follows that the proposed ACDC method can affect the uniformity of the wall thickness. The comparison between the experimental results and the *t*-test indicates ACDC can be effective for particularly strong curved part. However, ACDC also affects the non-uniformity of wall thickness in the straight/slight curved part of the substrate because of the flow direction change based on dripping. As one of the possibilities to improve this problem, the unit to gelate the solution on the substrate can be mounted directly above the PVA bath to change the solution from liquid to hydrogel.

These stereo dip-coating results indicate ACDC can contribute both thickness control and uniformity in the model. For example, the wall thickness of the real carotid artery is between 0.31 and 2.16 mm and the fabricated wall thickness could follow a realistic range of carotid arterial dimensions^32^. The other vessel parts can be also represented using a stereo dip-coating system with different substrates and withdrawal conditions if the thickness can be reproduced in the range of the system and the limitation of the dripping is solved.

The importance of curvature representation should be discussed. The curvature representation is a key factor of to reproduce the realistic blood vessels. As the described below, there is a limitation to represent the vessels with strong curvatures. However, there are no straight vessels in the human body and this curvature can influence the clinical technique while clinical trainings are performed. For example, the curvature of the substrate in this study is similar to that of femoral arteries^33^, and this artery is an important vessel for catheter treatment because the puncture for catheter insertion is performed at this vessel. For the realistic clinical training, this curvature can influence the clinical technique even if the curvature is not strong. If the clinical training is performed under the hemodynamic flow, the curvature can also influence the flow condition and the catheter behavior^3,34^. Therefore, the representation of the curvature can be an advantage of this model compared with the straight tube models and the regional representation of the realistic parts can also contribute to clinical trainings such as surgical treatment and first step of catheter treatment.

The limitation of dip-coating should be also discussed. There are difficulties for fabricating strong sharp curves and bifurcations. The strong sharp curves promote the high rotation speed of the substrate over the recognition speed in the images. In addition, control units of the substrate considering bifurcations have not been established.

## Conclusions

A stereo angular control dip-coating system has been developed and its characteristics have been elucidated in this study. The developed system has 5 degrees in rotational control accuracy from the vertical line of the liquid surface during substrate pull-up and the wall thickness was represented using the developed system in 50 mm/min of pull-up speed. The characteristics of the developed ACDC system indicate that ACDC is effective for fabricating the uniform wall thickness particularly in the strong curved parts. However, the wall thickness is non-uniform in the straight/slight curved parts because of the dripping based on the gravity and the rotation in the curved parts by ACDC. The real time gelation system should be developed for the next step.
